# Inertial and Magnetic Sensor Data Compression Considering the Estimation Error

**DOI:** 10.3390/s90805919

**Published:** 2009-07-24

**Authors:** Young Soo Suh

**Affiliations:** Department of Electrical Engineering, University of Ulsan, Namgu, Ulsan, Korea; E-Mail: yssuh@ulsan.ac.kr; Tel. +82-52-259-2196; Fax: +82-52-259-1686

**Keywords:** compression, estimation, inertial sensor, magnetic sensor

## Abstract

This paper presents a compression method for inertial and magnetic sensor data, where the compressed data are used to estimate some states. When sensor data are bounded, the proposed compression method guarantees that the compression error is smaller than a prescribed bound. The manner in which this error bound affects the bit rate and the estimation error is investigated. Through the simulation, it is shown that the estimation error is improved by 18.81% over a test set of 12 cases compared with a filter that does not use the compression error bound.

## 1. Introduction

Largely because of the MEMS technology, inertial sensors (accelerometers and gyroscopes) are becoming smaller and cheaper [1], which makes it possible to use inertial sensors in many applications. Inertial sensors are used in motion trackers [2], personal navigation systems [3] and remote control systems [4].

In some applications such as body motion trackers (for example, the product ‘Moven’ by XSENS), inertial sensors are used to track body movement. As the number of inertial sensors increases, the size of sensor data increases accordingly. The sensor data is transmitted to the microprocessor board through wired or wireless communication channels. In a wireless communication channel, the transmission speed is relatively low compared with a wired communication channel. The size of the sensor data needs to be reduced if it exceeds the capacity of the communication channel. For example, the transmission rate of raw sensor data for one MTx (commercial inertial and magnetic sensor) could be as high as 72 Kbps: 9 sensor outputs (3 accelerometers, 3 gyroscopes, and 3 magnetic sensors) × 500 Hz (maximum sampling rate) × 16 bits (16 bit A/D conversion for each sensor). If four MTx are used, the size of the sensor data exceeds the capacity of Zigbee (maximum rate is 250 Kbps [5]). More applications are expected as networked control and monitoring systems are becoming more popular [6].

One way to reduce the size of the sensor data is to compress the sensor data before transmission and decompress the received data in the microprocessor board. Data compression has been extensively studied in many areas [7]. In applications such as body motion trackers, real-time compression is preferable, in order to avoid delayed sensor data transmission and consequently the delay in motion estimation. One of the most popular real-time compression methods is ADPCM (Adaptive Differential Pulse Coded Modulation) [8], which is optimized for voice data. In [9], a simplified ADPCM method is used for inertial sensor data compression, where the maximum error (the difference between the original data and the compressed-and-then-decompressed data) is only relatively bounded (e.g., 1% of the sensor data).

The performance indices of data compression are the bit rate and the quality of compressed data. In voice data compression, the quality of compressed data is evaluated by listening to the compressed-and-then-decompressed voice data. This rather subjective evaluation makes sense since the final destination of compressed data is the human ear. On the other hand, the final destination of compressed inertial sensor data is usually a filter (such as a Kalman filter), where orientation is estimated. Thus the quality of compression should be judged by how the compression affects the estimation error.

In this paper, a modified ADPCM method is proposed, where the absolute maximum error bound is explicitly given. Also, we investigate how this error affects the estimation error. A part of this paper was presented in [10].

## 2. Inertial and Magnetic Sensor Data Compression and Estimation

**Figure 1.**
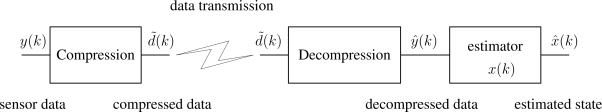
Overview of inertial and magnetic sensor data compression and estimation.

The overall process of compression and estimation is given in Figure 1, where *k* is a discrete time index. The objective is to estimate some states *x*(*k*) (attitude, heading, position, etc.) using inertial sensor data *y*(*k*) at a limited data transmission rate. The inertial sensor data *y*(*k*) is compressed into *d̃*(*k*) and transmitted to the microprocessor board. The compressed data is decompressed into *ŷ*(*k*) and the state *x*(*k*) is estimated using a filter.

Since the objective is to find a good estimator of *x*(*k*), the quality of compression is considered good if the estimation error *x*(*k*) − *x̂*(*k*) is small. The quality of the compression algorithm is evaluated using the following estimation error covariance:

(1) $$P_{\mathrm{error}}=E\{(x-\hat{x}(\hat{y}))\prime (x-\hat{x}(\hat{y}))\}$$

where *x̂*(*ŷ*) is an estimator when *ŷ* is used as an output.

Note that *P_error_* depends on the filter algorithm used to compute *x̂*(*ŷ*) in addition to the compression algorithm.

The ideal compression algorithm minimizes both *P_error_* and the bit rate. However, usually if *P_error_* is small, the bit rate tends to be large. In Section 4, we propose a compression algorithm where the maximum compression error is bounded. The maximum compression error bound plays a role of design parameter to adjust *P_error_* and the bit rate.

## 3. Modified ADPCM Algorithm

**Figure 2.**
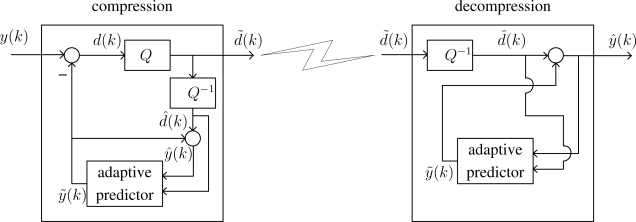
Encoder and decoder block scheme.

The ADPCM block scheme is given in Figure 2. We assume that *y*(*k*) is the output of *n_y_* bit uniform quantizer, where *y*(*k*) satisfies

(2) $$|y(k)|\;\leq \;y_{\max}$$

Let the quantization size *δ* of *y*(*k*) be defined by

(3) $$\delta =\frac{y_{\max}}{2^{n_{y}-1}}$$

If there is more than one sensor, we need one encoder for each sensor.

The sensor data *y*(*k*) is compared with the predictor output *ỹ*(*k*). The difference *d*(*k*) is coded into *d̃*(*k*) and this *d̃*(*k*) is transmitted to the estimator board. In the standard ADPCM, *d̃*(*k*) is a quantization index *i*(*k*). In this paper, *d̃*(*k*) consists of one bit mode information *m*(*k*) and a quantization index *i*(*k*):

(4) $$\tilde{d}(k)=\left[\begin{array}{c} m(k)\\ i(k) \end{array}\right]$$

In the decoder, the decompressed data is *ŷ*(*k*) = *ỹ*(*k*)+ *d̂*(*k*). The predictor output *ỹ*(*k*) can be computed from *d̂*(*k*) and thus does not need to be transmitted.

The adaptive predictor uses the same pole-zero configuration as that in CCITT G.726 ADPCM, which is an ADPCM speech compressor/decompressor protocol proposed in 1990 [11] :

(5) $$\tilde{y}(k)=\sum_{i=1}^{2}a_{i}(k-i)\hat{y}(k-i)+\sum_{i=1}^{6}b_{i}(k-1)\hat{d}(k-i)$$

From the assumption (2), if *ỹ*(*k*) *> y_max_* from (5), we set *ỹ*(*k*) = *y_max_*. Similarly, if *ỹ*(*k*) *<* −*y_max_*, we set *ỹ*(*k*) = −*y_max_*.

The adaptive algorithm in the G.726 protocol is used to adjust *a_i_* and *b_i_* and the detail is given in [11]; the tone and transition detector part was omitted since the part is only for voice data.

The compression error *e_c_*(*k*) is the difference between the original signal *y*(*k*) and the decompressed signal *ŷ*(*k*):

(6) $$\begin{array}{lll} e_{c}(k) & = & y(k)-\hat{y}(k)\\ & = & \left(\tilde{y}(k)+d(k))-(\tilde{y}(k)+\hat{d}(k)\right)\\ & = & d(k)-\hat{d}(k) \end{array}$$

Standard ADPCM algorithms [8] will be modified so that the maximum error is bounded as follows:

(7) $$|e_{c}(k)|\;\;\leq \;\;e_{\max}$$

Now *d̃*(*k*) coding is explained. The mode bit *m*(*k*) in *d̃*(*k*) is used to ensure (7). If the compression error *e_c_*(*k*) of a standard ADPCM method satisfies (7), then the mode is 0 (i.e., *m*(*k*) = 0). As will be seen in Section 3.1, this is true if *|d*(*k*)| *<* 2^*y_s_*(*k*)^*δ*, where *y_s_*(*k*) is an adaptive scaling factor. On the other hand, if the compression error *e_c_*(*k*) of a standard ADPCM method does not satisfy (7), then the mode is 1 (i.e., *m*(*k*) = 1) and a special uniform quantizer is used as in Section 3.2. Thus the mode *m*(*k*) is given by

(8) $$m(k)=\{\begin{array}{ll} 0, & \text{if}\frac{\left|d(k)\right|}{2^{y_{s}(k)}\delta }\leq 1\\ 1, & \text{otherwise} \end{array}$$

where *y_s_*(*k*) is an adaptive scaling factor.

The quantization index *i*(*k*) is defined differently when *m*(*k*) = 0 and when *m*(*k*) = 1.

### 3.1. Quantization index when m(k) = 0

If *m*(*k*) = 0, a signal *d*(*k*) is quantized with *n_d_* bits with a logarithm quantizer with an adaptive scaling factor *y_s_*(*k*), where the quantized index *i*(*k*) (1 ≤ |*i*| ≤ 2^*n_d_*−1^) satisfies

(9) $$f_{i-1}<\frac{\left|d(k)\right|}{2^{y_{s}(k)}\delta }\leq f_{i}$$

The sign of the index *i*(*k*) is the same as that of *d*(*k*). If *d*(*k*) = 0, then *i* = 1.

Coefficients *f_i_* in (9) are computed from *μ* law [8] so that *f_i_* satisfies the following:

$$\frac{{\text{log}}_{e}\left(1+\mu \left|f_{i}\right|\right)}{{\text{log}}_{e}(1+\mu )}=\frac{i}{2^{n_{d}}-1}$$

In this paper, *μ* = 5 is used and *f_i_* values for the case of *n_d_* = 5 is given in Table 1:

The scaling adaptation factor *y_s_*(*k*) is computed similarly with the standard ADPCM algorithm except that *y_s_*(*k*) is bounded as follows:

(10) $$3\leq y_{s}(k)\leq {\bar{y}}_{s}$$

We note that *ȳ_s_* is chosen so that (7) is satisfied. First we are going to derive the upper bound of *e_c_*(*k*) when *ȳ_s_* is given.

The decompressed signal *d̂*(*k*) is computed as follows:

$$\hat{d}(k)=\text{sgn}(i(k))2^{y_{s}(k)}\delta \frac{f_{i-1}+f_{i}}{2}$$

where

$$\text{sgn}(\alpha )=\{\begin{array}{ll} 1, & \alpha >0\\ 0, & \alpha =0\\ -1, & \alpha <0 \end{array}$$

The error *e_c_*(*k*) is then

(11) $$\begin{array}{lll} \left|e_{c}(k)\right| & = & \left|d(k)-\hat{d}(k)\right|\\ & \leq & 2^{y_{s}(k)}\delta \frac{f_{i}-f_{i-1}}{2} \end{array}$$

From the fact that *f_i_* is monotonically increasing and (10), we have

$$\begin{array}{lll} \left|e_{c}(k)\right| & \leq & 2^{y_{s}(k)}\delta \frac{f_{2^{n}d^{-1}}-f_{2^{n}d^{-1}}{}_{-1}}{2}\\ & \leq & 2^{{\bar{y}}_{s}}\delta \frac{f_{2^{n}d^{-1}}-f_{2^{n}d^{-1}}{}_{-1}}{2} \end{array}$$

Given *e_max_*, to satisfy (7), *ȳ_s_* should satisfy the following

(12) $$2^{{\bar{y}}_{s}}\delta \frac{f_{2^{n}d^{-1}}-f_{2^{n}d^{-1}}{}_{-1}}{2}\leq e_{\max}$$

Thus if *ȳ_s_* is chosen to satisfy (12), the quantization error is always smaller than *e_max_* when *m*(*k*) = 0. We also note that in addition to the global bound *e_max_*, if index *i*(*k*) is known, we have a less conservative bound given in (11):

(13) $${\bar{e}}_{c}(k)=2^{y_{s}(k)}\delta \frac{f_{i}-f_{i-1}}{2}$$

This bound will be used later in the estimation problem.

### 3.2. Quantization index when m(k) = 1

From (8), *m*(*k*) = 1 if *|d*(*k*)| *>* 2^*y_s_*(*k*)^*δ*. If *m*(*k*) = 1, then the logarithm quantizer used cannot guarantee the maximum error (7). Noting that *d*(*k*) = *y*(*k*) − *ỹ*(*k*), we can see that the mode is 1 if the difference between the output *y*(*k*) and the predicted value *ỹ*(*k*) is large, which happens when the signal change is not smooth but instead rather abrupt.

**Figure 3.**
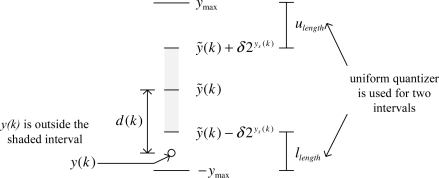
If *|y*(*k*) − *ỹ*(*k*)| > 2^*y_s_*(*k*)^*δ*, then *m*(*k*) = 1.

To ensure the maximum error condition (7), we introduce a uniform quantizer when the mode is 1. An example is given in Figure 3, where *y*(*k*) is outside the [*ỹ*(*k*) − *δ*2^*y_s_*(*k*)^*, ỹ*(*k*) + *δ*2^*y_s_*(*k*)^] interval and the mode is 1. The upper and lower intervals of the mode 1 interval (the mode 0 interval is the shaded area) are quantized with a uniform quantizer (quantization size is 2*e_max_*). The formal definition of the index in mode 1 is given as follows. Let *u_length_* and *l_length_* be defined by

$$\begin{array}{lll} u_{\mathrm{length}} & = & y_{\max}-\left(\tilde{y}(k)+\delta 2^{y_{s}(k)}\right)\\ l_{\mathrm{length}} & = & \left(\tilde{y}(k)-\delta 2^{y_{s}(k)}\right)+y_{\max} \end{array}$$

Let *u_level_* (the number of quantization levels for the upper interval *u_length_*) be defined by

$$u_{\mathrm{level}}=\{\begin{array}{ll} 0, & \text{if}\;u_{\mathrm{length}}\leq 0\\ \text{ceil}\left(\frac{u_{\mathrm{length}}}{2e_{\max}}\right), & \text{otherwise} \end{array}\mathrm{where}$$

ceil(*α*) is the smallest integer no smaller than *α*.

The index *i*(*k*) is given by

(14) $$i(k)=\{\begin{array}{ll} \text{floor}\left(\frac{y(k)-\tilde{y}(k)+\delta 2^{y_{s}(k)}}{2e_{\max}}\right) & \text{if}\;y(k)>\tilde{y}(k)+\delta 2^{y_{s}(k)}\\ u_{\mathrm{level}}+\text{floor}\left(\frac{\tilde{y}-\delta 2^{y_{s}(k)}-y(k)}{2e_{\max}}\right) & \text{if}\;y(k)<\tilde{y}(k)-\delta 2^{y_{s}(k)} \end{array}$$

where floor(*α*) is the largest integer no larger than *α*.

The decomposed signal *d̂*(*k*) is computed as follows.

$$\hat{d}(k)=\{\begin{array}{ll} \text{max}\left\{y_{\max},\tilde{y}(k)+\delta 2^{y_{s}(k)}+e_{\max}+i(k)2e_{\max}\right\}, & \text{if}\;i<u_{\mathrm{level}}\\ \text{min}\left\{-y_{\max},\tilde{y}(k)-\delta 2^{y_{s}(k)}-e_{\max}-\left(i(k)-u_{\mathrm{level}}\right)2e_{\max}\right\}, & \text{otherwise} \end{array}$$

Since a uniform quantizer is used, the compression error *e_c_*(*k*) in mode 1 is bounded by

(15) $$\left|e_{c}(k)\right|\leq {\bar{e}}_{c}(k)=e_{\max}$$

Note that the number of bits for the index *i*(*k*) is given by

$$n_{i}\;(k)=\text{ceil}\;\left({\text{log}}_{2}\;\left(\text{ceil}\;\left(\frac{u_{\mathrm{length}}}{2e_{m}}\right)+\text{ceil}\;\left(\frac{l_{\mathrm{length}}}{2e_{m}}\right)\right)\right)$$

When *m*(*k*) = 1, *n_i_*(*k*) changes depending on *ỹ*(*k*) and *y*(*k*). Note that when *m*(*k*) = 0, *n_d_* (the number of bits for *i*(*k*)) is constant. Also note that *n_i_* can be computed in the estimation board and thus does not need to be transmitted.

## 4. Kalman Filter Compensating the Compression Error

In Section 3, a compression method is proposed, where the maximum compression error is *e_max_*. Also if *m*(*k*) and *i*(*k*) are known, bounds of the compression error are given by (13) and (15). In this section, we use this information in a Kalman filter.

We assume that *y*(*k*) is generated by a linear system

(16) $$\begin{array}{rrr} x\left(k+1\right) & = & Ax(k)+w(k)\\ y(k) & = & Cx(k)+u(k) \end{array}$$

where *x ∈ R^n^* is the state, *y ∈ R^p^* is the output, and *w*(*k*) and *v*(*k*) are uncorrelated, zero-mean, white Gaussian noises that satisfy

$$\begin{array}{ll} E\{w(k)w^{\prime }(k)\}=Q, & E\{v(k)v^{\prime }(k)\}=R \end{array}$$

In the standard Kalman filter, *x*(*k*) is estimated using *y*(*k*). If *y*(*k*) is compressed, *ŷ*(*k*) = *y*(*k*) −*e_c_*(*k*) is used instead. From (13) and (15), *ē_c_*(*k*) can be computed, which is used to reduce the estimation error. If we assume *e_c,i_*(*k*) (*i*-th element of *e_c_*(*k*)) has a uniform distribution, $E\left\{e_{c,i}^{2}(k)\right\}=\frac{1}{3}{\bar{e}}_{c,i}^{2}(k)$. By treating the compression error *e_c_*(*k*) as measurement noise in *y*(*k*), the following model can be used for an estimator.

(17) $$\begin{array}{rrr} x\left(k+1\right) & = & Ax(k)+w(k)\\ \hat{y}(k) & = & Cx(k)+\bar{v}(k) \end{array}$$

where

(18) $$\bar{R}(k)=E\{\bar{v}(k){\bar{v}}^{\prime }(k)\}=R+\frac{1}{3}\left[\begin{array}{lll} {\bar{e}}_{c,1}^{2}\;(k) & & \\ & \ddots & \\ & & {\bar{e}}_{c,p}^{2}\;(k) \end{array}\right]$$

Since the compression error is compensated in the estimation algorithm, we can expect a smaller estimation error, which is verified through the simulations in Section 5. When a Kalman filter is used for (17), the estimation error covariance *P*(*k*) = E{(*x*(*k*) − *x̂*(*k*))(*x*(*k*) − *x̂*(*k*))′} can be computed from a Riccati equation [12].

(19) $$P(k+1)=AP(k)\;A^{\prime }+Q-AP(k)C^{\prime }{\left(CP(k)C^{\prime }+\bar{R}(k)\right)}^{-1}CP(k)\;A^{\prime }$$

Since *R̄*(*k*) depends on *ŷ*(*k*), we cannot compute *P*(*k*) before simulation. To evaluate the estimation error covariance without simulation, we use an upper bound of *R̄*(*k*):

$$\bar{R}(k)\leq R+\frac{1}{3}\left[\begin{array}{lll} e_{\max,1}^{2} & & \\ & \ddots & \\ & & e_{\max,p}^{2} \end{array}\right]={\bar{R}}_{\max}$$

Using this *R̄_max_* in place of *R̄*(*k*), we can find a steady-state solution to (19).

(20) $$\bar{P}=A\bar{P}A^{\prime }+Q-A\bar{P}C^{\prime }{\left(C\bar{P}C^{\prime }+{\bar{R}}_{\max}\right)}^{-1}C\bar{P}A^{\prime }$$

*P̄* can be considered as an upper bound of *P*(*k*) in (19). From *P̄*, we can see how *e_max,i_* affects the estimation error.

A similar idea is used in [13], where a networked estimation problem is considered.

## 5. Simulation

We compared three data sets using the proposed compression algorithm. Original data is 1600 bits/s for each sensor : 16 bit A/D converted data (i.e., *n_y_* = 16) with the sampling rate being 100 Hz. We used *n_b_* = 5: that is, the number of bits for the quantization index when *m*(*k*) = 0 is 5. *e_max_* for each sensor is chosen so that *e_max_* = 300*δ*, where *δ* is different for each type of sensors. *ȳ_s_* = 12 is found to satisfy (12).

Bit rates for the three compressed data sets are given in Table 2. All three data sets are obtained using XSENS MTx (3 accelerometer, 3 gyroscopes, and 3 magnetic sensors). Holding MTx with a hand, we moved MTx slowly (data set 1) and fast (data set 2). Data set 3 is obtained from a personal navigation system, where MTx is attached on the shoe of a pedestrian [3].

The bit rates of data set 3 is the largest because the change of data is the most abrupt. In particular, when the shoe contacts the floor, there is a large change in the accelerometer and gyroscope outputs, and consequently the compression algorithm becomes *m*(*k*) = 1 more often.

To see how the compression error affects the estimation error, a simple one dimensional attitude estimation problem is considered. An attitude (*θ*) is estimated using two outputs: *y_i_* is an inclinometer output and *y_g_* is a gyroscope output, where

(21) $$\begin{array}{lll} y_{i} & = & \theta +v_{i}\\ y_{g} & = & \dot{\theta }+v_{g} \end{array}$$

where *v_i_* and *v_g_* are measurement noises and $q_{i}=E\left\{v_{i}^{2}\right\}=0.13\times 10^{-1}$ and $q_{g}=E\left\{v_{g}^{2}\right\}=0.78\times 10^{-5}$.

An indirect Kalman filter ([12]) is used to estimate *θ* using *y_i_* and *y_g_*. In the indirect Kalman filter, the gyroscope output is integrated to compute *θ_i_*, i.e.,

$${\dot{\theta }}_{i}=y_{g}$$

Defining *θ_δ_* as

$$\theta_{\delta }=\theta_{i}-\theta$$

we obtain

(22) $${\dot{\theta }}_{\delta }=v_{g}$$

In the indirect Kalman filter, *θ_δ_* is first estimated and *θ̂* is obtained indirectly from *θ̂ = θ_i_* − *θ̂_δ._*

By discretizing (22) with the sampling period *T* (*T* = 0.01*sec*), we have

(23) $$\begin{array}{lll} \theta_{\delta }(k+1) & = & \theta_{\delta }(k)+\sqrt{T}v_{g}\\ \theta_{i}-y_{a} & = & \theta_{\delta }(k)-v_{i} \end{array}$$

**Figure 4.**
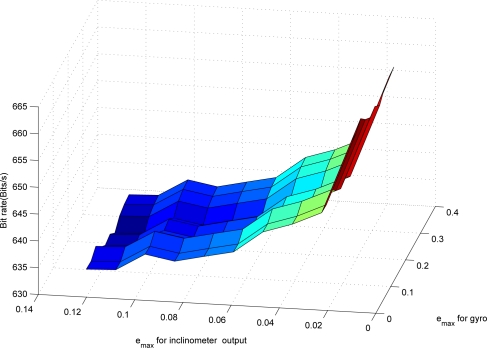
Relationship between *e_max_* and the bit rate.

Now the proposed compression algorithm is used to compress *y_i_* and *y_g_*. The maximum compression errors *e_max,i_* (*e_max_* for *y_i_*) and *e_max,g_* (*e_max_* for *y_g_*) affect both the bit rate and the estimation error. If *e_max_* is small, the chance that the mode becomes 1 increases. Thus the bit rate becomes large. How the bit rate changes with the changing *e_max_* is given in Figure 4. The data *y_i_* and *y_g_* are generated using Matlab.

From Figure 4, we can see that the bit rate of the inclinometer outputs increases rapidly as *e_max,i_* is decreased. On the other hand, the bit rate of the gyroscope outputs does not change much as *e_max,g_* is decreased. The bit rate depends on how often the mode becomes 1: note that *n_i_* (the number of bits needed for the quantized data when the mode is 1) is generally larger than *n_d_* (the number of bits needed when the mode is 0). If the original signal is sufficiently smooth, *d*(*k*) is small since the predicted value *ỹ*(*k*) is very close to *y*(*k*). Thus even if we decrease *e_max_*, *d*(*k*) still satisfies *|d*(*k*)| *≤* 2^*y_s_*(*k*)^*δ* condition in (8).

**Figure 5.**
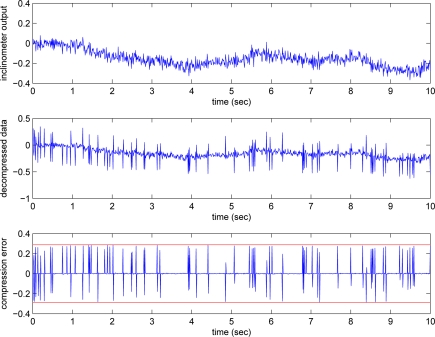
Inclinometer output, decompressed data, and error.

**Figure 6.**
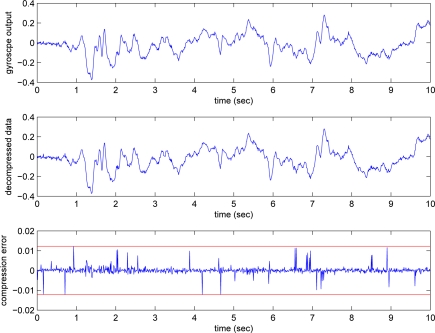
Gyroscope output, decompressed data, and error.

The *y_i_* and *y_g_* signals and the compressed signals are given in Figures 5 and 6, where *e_max,i_* = 0.2876 and *e_max,g_* = 0.0122. We can see that the gyroscope output is relatively smooth compared with the inclinometer output. Thus the bit rate of the gyroscope output is relatively insensitive to the changes in *e_max,g_*.

**Figure 7.**
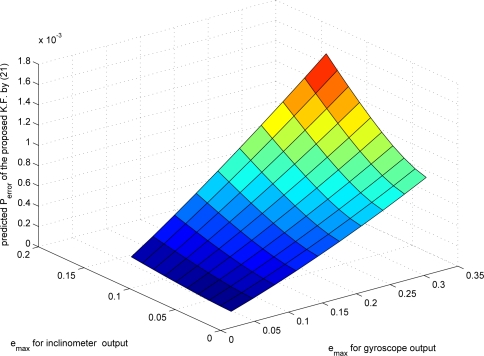
Predicted estimation error (*P̄*) of the proposed filter.

**Figure 8.**
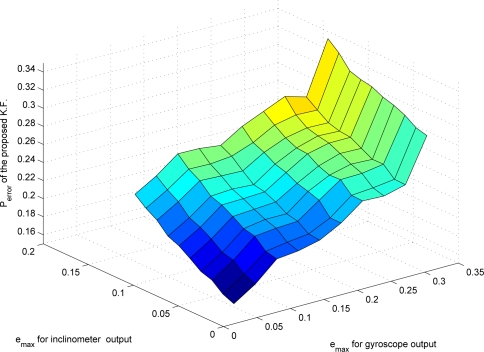
Estimation error $\left(\sqrt{\sum {\left(\theta (k)-\hat{\theta }(k)\right)}^{2}}\right)$ of the proposed filter.

The effects of changes in *e_max_* on the estimation error are given in Figure 7, where the estimation error is predicted using (20). Actual estimation error from simulation is given in Figure 8, where $\sqrt{\sum {\left(\theta (k)-\hat{\theta }(k)\right)}^{2}}$ is computed.

**Figure 9.**
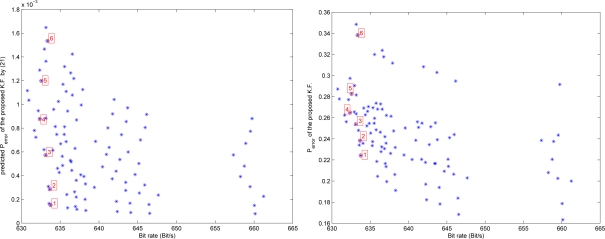
Bit rates and *P_error_*: predicted value by (21) and the experiment result.

The relationship between bit rates and estimation error is presented in Figure 9, where data are from Figures 4, 7 and 8. In the left graph, the points 1–6 have similar bit rates but different *P_error_*. Thus in the following simulation, we chose the point 1, which corresponds to *e_max,i_* = 0.2876 and *e_max,g_* = 0.0122. We compared three different filters: (a) a standard Kalman filter using *y_i_* and *y_g_*; (b) a Kalman filter using *ŷ_i_* and *ŷ_g_* with compression error compensation (proposed in Section 4); (c) a Kalman filter using *ŷ_i_* and *ŷ_g_* without compression error compensation. We randomly generated 12 data sets and the results are given in Table 3.

It is not surprising that the *P_error_* of the standard Kalman filter is the smallest because the original data *y_i_* and *y_g_* are used for measurements. We can also see that the *P_error_* of the proposed filter is smaller than that of the filter (c). On average, the estimation error of the proposed filter is smaller by 18.81% compared with that of the filter (c). Note that the proposed filter (b) and the filter (c) use the same decompressed data *ŷ_i_* and *ŷ_g_* for measurements. However, in the proposed filter, the compression error information (13) and (15) are explicitly used in (18), whereas they are ignored in the filter (c). In summary, the estimation error reduction was possible because of two facts: (1) the proposed compression method provides the compression error bound (13) and (15), and (2) the proposed filter algorithm explicitly uses the error compression bound.

## 6. Conclusion

In this paper, we have proposed a compression method for inertial and magnetic sensor data. The proposed compression method guarantees that the compression error is bounded by a prescribed *e_max_* value. Smaller *e_max_* value usually increases the bit rate and reduces the estimation error of the filter when the decompressed data is used. Thus *e_max_* plays the role of a trade-off parameter between the bit rate and the estimation error. Also we have seen that by using a bound on compression error, the estimation error can be reduced.

## Figures and Tables

**Table 1.** *f_i_* values for *n_d_* = 5.

*f*_0_	*f*_1_	*f*_2_	*f*_3_	*f*_4_
0	0.0237	0.0503	0.0799	0.1131
*f*_5_	*f*_6_	*f*_7_	*f*_8_	*f*_9_
0.1501	0.1915	0.2379	0.2899	0.3479
*f*_10_	*f*_11_	*f*_12_	*f*_13_	*f*_14_
0.4129	0.4855	0.5667	0.6575	0.7593
*f*_15_	*f*_16_			
0.8729	1.0000			

**Table 2.** Bit rates for 3 inertial and magnetic sensor data sets.

	accelerometers	gyroscopes	magnetic sensors

data set 1	643.2	641.6	617.6
	648.0	632.0	622.4
	656.0	628.8	614.4

data set 2	659.2	660.8	617.6
	662.4	640.0	622.4
	668.8	638.4	619.2

data set 3	771,2	715.2	638.4
	798.4	696.0	633.6
	694.4	686.4	627.2

**Table 3.** Bit rates and estimation error of 3 filters: (a) a standard Kalman filter with uncompressed data, (b) the proposed method with *ŷ_i_* and *ŷ_g_*, and (c) a Kalman filter with *ŷ_i_* and *ŷ_g_*.

experiment	Bit rate		*P_error_*			% improvement ((c) - (b)) / (c)
	*ŷ_g_*	*ŷ_i_*	(a)	(b)	(c)	
1	684.8	620.3	0.318	0.437	0.452	3.32
2	654.4	623.3	0.173	0.361	0.492	26.62
3	640.0	621.3	0.138	0.345	0.402	14.19
4	645.7	622.2	0.139	0.312	0.455	31.39
5	631.2	625.7	0.161	0.382	0.447	14.50
6	633.6	621.6	0.121	0.310	0.422	26.55
7	692.7	692.7	0.308	0.427	0.445	3.96
8	640.8	622.2	0.120	0.280	0.317	11.77
9	634.4	618.6	0.134	0.295	0.537	45.15
10	696.1	618.8	0.423	0.487	0.503	3.14
11	638.4	632.9	0.191	0.394	0.464	14.99
12	638.4	621.5	0.136	0.292	0.417	30.13
average	652.5	628.4	0.197	0.360	0.446	18.81
